# A Randomized Controlled Study to Compare the Efficacy of High Frequency Nasal Oxygenation with Conventional Oxygen Therapy for Postoperative Oxygenation in Patients Undergoing Exploratory Laparotomies

**DOI:** 10.4274/TJAR.2025.251895

**Published:** 2025-10-14

**Authors:** Geetanjali T. Chilkoti, Poonam Sehrawat, Medha Mohta, Michell Gulabani

**Affiliations:** 1 University College of Medical Sciences & Guru Teg Bahadur Hospital, Hospital, Clinic of Anaesthesiology, Delhi, India

**Keywords:** Hypoxemia, laparotomies, oxygen, postoperative period, patient comfort

## Abstract

**Objective:**

Postoperative pulmonary complication (PPC) is one of the leading causes of poor surgical outcome leading to longer hospital or intensive care unit stay and mortality especially with upper abdominal surgeries having long duration. High-frequency nasal oxygenation (HFNO) has recently been employed for postoperative oxygenation following extubation in surgical patients.

**Methods:**

Fifty consenting adult patients aged 18-65 years of either sex scheduled for exploratory abdominal surgeries under general anaesthesia (GA) with Assess Respiratory Risk in Surgical Patients in Catalonia score ≥26 i.e., moderate to high risk were enrolled. After instituting all routine the American Society of Anesthesiologists recommended monitoring, baseline haemodynamic parameters were recorded. Patients were preoxygenated with 100% oxygen and GA was administered as per standard institutional protocol. Following extubation, patients were randomly allocated into one of the groups comprising 25 patients each where Group C and Group H received conventional oxygen therapy via simple face mask and HFNO respectively. The FiO_2_ was titrated (from 45% to 100%) by the anaesthesiologist to maintain a SpO_2_ of 95% or more. Arterial blood samples were collected after extubation at various designated time points i.e. 2^nd^, 6^th^,12^th^ and 24^th^ hr, The P/F ratio, PaO_2_, PaCO_2_, SaO_2_/FiO_2_ ratio along with haemodynamic parameters, incidence of PPCs/acute hypoxemic respiratory failure (AHRF), atelectasis and comfort score were also recorded.

**Results:**

Significant improvement in all oxygenation parameters following the use of HFNO for postoperative oxygenation; however, PaCO_2_, haemodynamic variables, complications, incidence of PPCs/AHRF and atelectasis remained comparable between the two groups.

**Conclusion:**

Preventive use of HFNO for post operative oxygenation amongst moderate to high-risk patients scheduled for exploratory abdominal surgery improves oxygenation.

**Main Points**

• Reported incidence of postoperative pulmonary complications (PPCs) is as high as 40% following abdominal surgeries.

• Recently, high frequency nasal cannula (HFNC) has been increasingly used for providing postoperative oxygenation.

• Its definite role following major abdominal surgeries needs further exploration.

• We observed that the preventive use of HFNC for postoperative oxygenation amongst patients scheduled for exploratory abdominal surgery improves oxygenation; however, no difference in acute hypoxemic respiratory failure/PPCs, reintubation rate, Chest X-ray proven atelectasis and complications.

## Introduction

Postoperative pulmonary complications (PPCs) are known to be the second most common complication after surgery, with an incidence ranging from 2% to 19% in non-cardiac procedures and as high as 40% following abdominal surgeries.^1^ Hypoxemia is one of the most frequent PPCs, making postoperative oxygen administration essential.^2^

Low-flow conventional oxygen therapy (COT) continues to be the primary method for oxygen delivery in the postoperative period. However, high-frequency nasal oxygenation (HFNO) has recently been introduced due to its benefits, such as delivering a more predictable FiO_2_, improved humidification, reduced anatomical dead space, and greater patient comfort.^3^ Despite these advantages, HFNO failure in patients with pulmonary complications can result in delayed intubation, leading to increased morbidity and mortality.^4^ As a result, its safety and effectiveness are being more frequently studied in the literature, although the findings have been inconsistent.^5, 6^

Most of the studies evaluating the efficacy of HFNO for postoperative oxygenation have been conducted in obese patients and following cardio-thoracic surgeries.^7, 8, 9^ It’s role in routine surgical procedures like laparotomies with long surgical duration has not yet been extensively studied except a single large multicentric trial that evaluated the efficacy of HFNO along with lung-protective ventilation strategy for postoperative oxygenation following major abdominal surgeries but lacked the measurement of all blood oxygenation parameters.^2^ Additionally, it also did not incorporate any radiological method to rule out PPCs and quality of recovery (QoR).

Therefore, the present study was undertaken to evaluate the efficacy of HFNO compared with COT for prevention of PPCs in patients undergoing exploratory abdominal laparotomies during the immediate post-operative period. The primary outcome was arterial oxygen tension to inspiratory oxygen fraction partial pressure of oxygen (PaO_2_)/FiO_2_ (P/F) ratio at the end of day one between the two groups. Various secondary outcomes included the PaO_2_, arterial oxygen saturation (SaO_2_) SaO_2_/FiO_2_ (S/F) ratio, partial pressure of carbon dioxide (PaCO_2_), incidence of PPCs and complications at various time points in the first 24 hours postoperatively.

## Methods

This prospective randomised controlled study was conducted in a tertiary care centre following approval from the Institutional Ethics Committee of Human Research (IEC-HR) University College of Medical Sciences, University of Delhi (approval no.: IECHR-2022-55-71, date: 30.08.2022) and subsequently registered under the Clinical Trials Registry-India (CTRI) with number (CTRI/2023/01/049207).

A written informed consent from each participant was taken prior to their recruitment. The study was carried out in accordance with the principles of the Declaration of Helsinki 2013 and confirmed the use of patient data for research and educational purposes. Fifty consenting adult patients aged 18-65 years of either sex scheduled for exploratory abdominal surgeries under general anaesthesia (GA) with Assess Respiratory Risk in Surgical Patients in Catalonia score ≥26 i.e., moderate to high risk^10^ were enrolled. Patients with a history of cardiac disease, restrictive or obstructive pulmonary disease or asthma causing functional limitations, body mass index >40 kg m^2-1^, inability to comprehend oral or written information were excluded from the study.

In the operating room, all routine the American Society of Anesthesiologists (ASA) recommended mandatory monitoring were instituted and baseline haemodynamic parameters i.e. heart rate (HR), systolic blood pressure (SBP), diastolic blood pressure (DBP) and mean blood pressure were recorded. Patients received normal tidal volume preoxygenation with 100% oxygen for three minutes. GA was administered as per standard institutional protocol and trachea was intubated using an endotracheal tube (ETT). At the end of surgery, patients were randomised into one of the two groups namely C and H using a computer-generated random number table and allocation concealment was done by using sequentially numbered opaque sealed envelopes. Following adequate reversal of neuromuscular blockade, ETT was removed, and patients were shifted to the post-anaesthesia care unit (PACU). Patients in group C received COT via simple face mask, whereas those in group H, HFNO via high frequency nasal cannula (HFNC) with flow rate of 35 to 60 L min for 24 hours.

The FiO_2_ was titrated (from 45% to 100%) by the anaesthesiologist to maintain an SpO_2_ of 95% or more in both the groups and patients were observed for a period of three days. Standard bedside multipara monitor was used to record the SpO_2_, non-invasive blood pressure and HR every 15 minutes for the first 2 hours. Arterial blood gas (ABG) samples were collected in the PACU, at various designated time points i.e. at the end of 2^nd^, 6^th^, 12^th^ and 24^th^ hour postoperatively. Additional oxygen was continued beyond 24 hours of the study, if SpO_2_ continued to be below 93% after oxygen discontinuation.

The duration and incidence of SpO_2 _<90% was noted. The incidence of hypoxemia (defined as P/F ratio £ 300 mmHg) was recorded. The management and further ABG sampling were as per the discretion of anaesthesiologist. The differences of PaO_2_, PaCO_2_, P/F and S/F ratios, were noted and compared between the groups.

The proportion of patients who developed PPCs, such as pneumothorax, pleural effusion or suspected pneumonia (defined by at least one of the following criteria: new or changed sputum, new or altered Chest X-ray findings, oral temperature >38.3 °C, and white blood cell count > 12×10^9^/L) and atelectasis (as evident on Chest X-ray), were also recorded.^2^ Additionally, the proportion of patients who developed acute hypoxemic respiratory failure (AHRF) in both groups was documented. It was defined by meeting any one of the hypoxemic criteria (SpO_2 _<92% while on at least 10 L min oxygen, PaO_2 _<60 mmHg on room air, or PaO_2 _<80 mmHg with supplemental oxygen), along with at least one of the following signs: respiratory rate >25 breaths min, dyspnoea with use of accessory muscles and ABG finding i.e. respiratory acidosis with pH <7.30 or PaCO_2_ >50 mmHg. The incidence of non-invasive ventilation (NIV) requirement and reintubation, if any, was also noted and compared between the two groups.

Chest X-ray was done as baseline and at the end of day three, any findings suggestive of atelectasis i.e. small volume linear shadows either peripherally or at lung bases, opacification of the lung along with mediastinal shift, elevation of ipsilateral diaphragm, rib crowding etc. was recorded. The Chest X-ray was reported by a senior radiologist who remained blinded to the group allocation. The adverse effects related to HFNC application and COT e.g. throat or nasal pain, air leak, and abdominal distension were also recorded.

Patients were asked to rate the effect of the treatment on their comfort using the following scale: 1 (very poor), 2 (poor), 3 (sufficient), 4 (good), and 5 (very good).^11^ Additionally, patient satisfaction scores, Chest X-ray confirmed PPCs, the proportion of PPCs, AHRF, reintubation rates, NIV use, and complications related to the intervention (such as air leaks, nasal or throat pain, and abdominal distension) were also documented.

### Statistical Analysis

The sample size calculation was done based on a pilot study of ten patients, where the postoperative P/F ratio on day one was observed to be 356 mmHg [standard deviation (SD) 40.2] with COT in patients undergoing exploratory laparotomy. Considering this SD, a difference of 10% was considered as significant with the alpha error of 0.05 and 80% power of study, a sample size of 40 patients with 20 in each group was calculated. Further, considering a dropout rate of 10%, the final sample size of 50 with 25 patients in each group was decided.

The data was analysed using the statistical software version 20. Quantitative variables were reported with mean (SD) or median [interquartile range] and qualitative variables with number and percentage. The normality was tested using a box-whisker plot and the statistical test Shapiro-Wilk test. The unpaired student t-test was applied for normally distributed variables. The chi-square and Fisher’s exact test were applied for the qualitative variables. Linear mixed model with a suitable covariance structure was performed to compare the hemodynamic variables followed by Bonferroni correction to control type I error. The *P *value less than 0.05 was considered as statistically significant.

## Results

**Figure 1:**
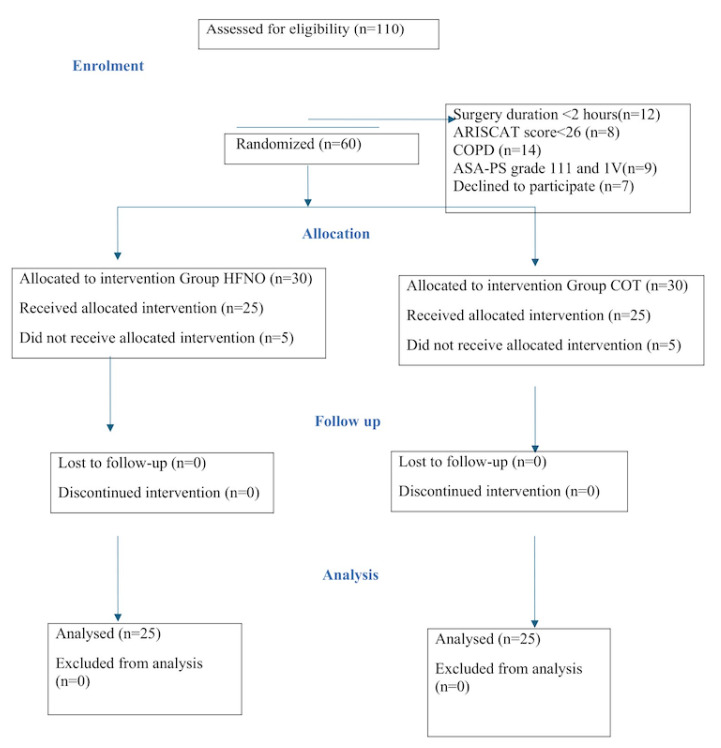
Consort flow diagram of 50 patients who received treatment in two groups, C and H respectively. Group COT, conventional oxygen therapy; Group HFNO, high frequency nasal oxygenation; COPD, chronic obstructive pulmonary disease; ASA-PS, the American Society of Anaesthesiologist Physical Status.

A total of 110 patients were enrolled; out of which 50 were excluded (Figure 1). Hence, 60 patients were randomised into two groups with 30 in each. Further, five patients in each group were excluded as they required elective mechanical ventilation. Finally, 50 patients with 25 in each group were included.

The demographic parameters i.e. patient’s age, weight, gender and ASA physical status were comparable in both groups. Similarly, the duration of surgery and baseline haemodynamic parameters were also comparable between the two groups (Table 1).

The mean P/F ratio at the 24^th^ hour was found to be significantly higher in group H compared to group C i.e. 690.80±79.35 vs. 312.82±29.66, respectively. An unpaired t-test was performed to compare the ratio between the groups and homogeneity of variance assumption was tested using Levene’s test, which showed a violation of the normality assumption. So, Welch’s corrected *P *value was applied. The mean difference was 377.98 (343.40 to 412.55) i.e. group H ratio was 378 units higher than group C (*P *value: Welch test <0.001). The average P/F ratio between the groups at each observed time point was significantly higher in group H (*P *value: Bonferroni correction <0.001) (Table 2). Similarly, the difference in the incidence of hypoxemia at all designated time points was significant [16% (n = 4) in group C vs. none in H].

Comparison of PaO_2_ between the groups at different time points revealed a significant difference in the estimated marginal mean difference (HFNO minus COT at 95% confidence interval) showing higher values in group-H (*P *< 0.001) (Table 3). Similarly, the S/F ratio on comparison showed higher readings at all designated time intervals in the group-H (*P* value of estimated marginal mean *P *< 0.001). The PaCO_2 _and haemodynamic variables (SBP, DBP, MAP, HR) between the two groups at all designated time points were comparable. The median patient comfort score was elevated with the group H vs group C i.e. 4.0 [3.5-4.0] vs 3.0 [2.0-3.0] (*P *< 0.001) respectively (Table 4).

Five patients in group C had complaints of nasal/throat pain and two patients complained of air leak. Four patients in group H complained of abdominal distension for which the airflows were adjusted accordingly, following which patients were relieved. Two patients in the group C and one in the group H developed pneumonia postoperatively (Table 4). All these three patients were managed on broad-spectrum antibiotics in the ward and discharged later.

As far as the Chest-X-ray proven PPCs are concerned, two patients in group C and one in H were diagnosed to have atelectasis (Table 3). None of them in either of the groups developed AHRF, neither required NIV or reintubation nor was there any mortality amongst included patients. Therefore, the complications associated with the interventions, Chest-X-ray proven atelectasis, PPCs, AHRF, reintubation, and mortality between the two groups were comparable (Table 3).

## Discussion 

In the present study we observed a significant improvement in all oxygenation parameters following the use of HFNC for postoperative oxygenation; however, PaCO_2_, haemodynamic variables, complications, incidence of PPCs/AHRF and atelectasis remained comparable between the two groups.

HFNO has an edge over COT by virtue of its convenient application, providing heated and humidified oxygen at high flows and positive end-expiratory pressure thus improving oxygenation in addition to enhanced patient comfort.^12, 13^ Amidst the widespread clinical benefits of HFNO, it has become increasingly popular for postoperative oxygenation having been successfully employed for preventing PPCs in obese patients^7, 8^ and following cardio-thoracic surgical procedures.^14^ The risk factors for PPCs are multifactorial, the most common amongst the surgery-related risk factors include upper abdominal procedures accompanied by a long duration.^15^ The use of HFNO for postoperative oxygenation in this subset of patients, to prevent hypoxemia and development of PPCs remains an avenue that has not been extensively studied.

The popular OPERA trial is the only clinical study to have evaluated the effectiveness of HFNO compared with COT for postoperative oxygenation in patients undergoing upper abdominal surgeries.^2^ The primary outcome was the proportion of patients developing hypoxaemia which was defined as P/F ratio ≤300, one hour after tracheal extubation. The study did not report any significant reduction in the incidence of postoperative hypoxaemia with HFNO. This result contrasts our study, possibly due to a few reasons. The primary end point in OPERA trial was arbitrary (as reported by them also) which may not have reflected disease severity. The oxygenation parameters were neither measured beyond one hour, nor any radiological method was employed to rule out PPC’s. Additionally, the duration of HFNC application in the OPERA trial varied between patients in the postoperative period with a median duration of 15 hours versus 24 hours uniformly for all patients in the present study.

Amongst the available studies evaluating HFNO for postoperative oxygenation, there has not been any defined recommended time duration for its administration. However, a study recently highlighted that its application for 24 hours after tracheal extubation has been found to be sufficient to reduce the re-intubation rate.^16^

The OPERA study aimed to explore the efficacy of HFNO along with intraoperative lung-protective ventilation on postoperative oxygenation. The use of two preventive strategies simultaneously to reduce PPCs could have confounded the results of their study. Further, in their research, no attempt was made to ensure that patients administered HFNC had their mouth closed, which could have led to reduced airway pressure unlike in the present study where closed mouth breathing was ensured in patients receiving HFNO. Previous studies have shown that the high flowrates in HFNO translate into clinically significant airway pressures only when the mouth is closed.^17, 18^ These aforementioned factors in the OPERA trial could have perhaps led to an insignificant reduction in the incidence of postoperative hypoxemia with the preventive application of HFNO in patients scheduled for abdominal surgery.

We observed a higher comfort score with the HFNO as opposed to the OPERA trial in which comparable results were obtained.^2^ In the present study, the complications associated with the interventions namely Chest-X-ray proven atelectasis, PPCs, AHRF, reintubation, and mortality between the two groups were comparable. Our results contrast with the conclusion of a meta-analysis where reintubation rate was observed to be significantly reduced with the use of HFNC; however, like our study the incidence of PPCs and mortality were comparable.^19^ Additionally, no difference was observed in haemodynamic parameters between the two groups post operatively, like the OPERA trial.^2^

We determined the sample size based on the P/F ratio as the study aimed to evaluate the efficacy of HFNO for postoperative oxygenation; however, for complications such as PPC, AHRF and reintubation rates, study may not have adequate power. Amongst complications, Chest-X-ray to rule out basal atelectasis was done on day 3. The decision to perform the imaging on day 3 is based on the previous studies which have shown that the high incidence of PPC is seen within 72 hrs.^20, 21^

The present study is dealt with few limitations. Firstly, we did not record the long-term outcomes such as length of hospital stay and morbidity. Secondly, a patient-related outcome measure using QoR score could have been assessed. Thirdly, blinding of the treatment arm was not feasible due to the nature of intervention and thus could have been a source of observer bias. Finally, more sensitive, non-ionizing imaging modalities, such as bedside ultrasonography is now the standard tool to detect postoperative atelectasis but not be attempted due to logistic constraints.

### Future Recommendations

A well-designed randomized controlled trial is needed to validate the findings of the current study. In addition, studies utilizing complications (e.g. PPCs, AHRF, reintubation rates etc.) and long-term outcomes (e.g. total hospital stay, morbidity etc.) as primary outcomes, in order to confirm the benefits of HFNO are warranted.

## Conclusion

It was observed that the preventive use of HFNO for post operative oxygenation amongst moderate to high-risk patients scheduled for exploratory abdominal surgery improves oxygenation; however, the incidence of AHRF/PPCs, reintubation rate, Chest-X-ray proven atelectasis, patient comfort score and adverse events remained comparable between the two groups.

## Ethics

**Ethics Committee Approval:** Ethical approval was obtained from the Institutional Ethics Committee of Human Research (IEC-HR) University College of Medical Sciences, University of Delhi (approval no.: IECHR-2022-55-71, date: 30.08.2022).

**Informed Consent:** A written informed consent from each participant was taken prior to their recruitment.

## Figures and Tables

**Table 1. Demographic Data of Patients with Preoperative Clinical Characteristics and Duration of Surgery According to the Groups:** 

**Variable name**	**Group H (n = 25)**	**Group C (n = 25)**	**Mean difference (Group H minus Group C) 95% CI**	***P* value**
Age (years) - Mean (SD)	36.28±12.10	36.48±15.54	-0.20 (-8.12 to 7.72)	0.960
Gender				
Male	17 (68.0)	15 (60.0)	-	0.556
Female	8 (32.0)	10 (40.0)	-	
ASA PS				
ASA1	17 (68.0)	14 (56.0)	-	0.382
ASA2	8 (32.0)	11 (44.0)	-	
Weight (kg)	72.24±12.49	68.56±9.29	3.68 (-2.74 to 10.10)	0.255
Duration of surgery (minutes)	316.8±56.18	348.0±54.77	-31.20 (-62.75 to 0.351)	0.053
SBP baseline	130.76±9.27	134.04±12.04	-3.28 (-9.39 to 2.83)	0.286
DBP baseline	84.12±7.44	86.28±7.67	-2.16 (-6.46 to 2.14)	0.317
MBP baseline	99.68±7.50	102.98±7.91	-2.40 (-6.79 to 1.99)	0.277
HR baseline	102.88±12.54	108.64±13.62	-5.76 (-13.2 to 1.69)	0.126

Group C, conventional oxygen therapy; Group H, high frequency nasal oxygenation; SBP, systolic blood pressure; DBP, diastolic blood pressure; ASA PS, American Society Anaesthesiologists Physical Status; CI, confidence interval; MBP, mean blood pressure; HR, heart rate; SD, standard deviation

**Table 2. Mean P/F ratio and P/F Ratios at Different Time Intervals Between the Groups:** 

**Variable name** **(P/F ratios at different time intervals) **	**Mean (SD)**		**Estimated marginal mean **		**Estimated marginal mean difference (HFNO minus COT) (95% CI) **	***P *value (with Bonferroni correction) **
	**Group H (n = 25) **	**Group C (n = 25) **	**Group H (n = 25) **	**Group C (n = 25) **		
P/F: 2^nd^ hr	683.4±76.62	310.0±31.70	683.4 (11.83)	310.0 (11.83)	373.38 (340.02 to 406.75)	< 0.001^#^
P/F: 6^th^ hr	702.6±82.1	308.99±36.51	702.6 (11.83)	308.99 (11.83)	393.61 (360.24 to 426.97)	< 0.001^#^
P/F: 12^th^ hr	702.1±89.73	313.16±28.81	702.1 (11.83)	313.59 (11.83)	388.94 (355.58 to 422.31)	< 0.001^#^
P/F: 24^th^ hr	690.8±79.35	312.86±29.64	690.8 (11.83)	312.86 (11.83)	377.94 (344.58 to 411.31)	< 0.001^#^

Data are shown as mean±SD; Group C, conventional oxygen therapy; Group H, high frequency nasal oxygenation; P/F ratio, PaO_2_/FiO_2_; *P *< 0.05, statistically significant^#^; hr, hour; CI, confidence interval; SD, standard deviation

**Table 3. Partial Pressure of Oxygen (PaO2) Between the Groups:** 

**Variable (PaO_2_) time**	**Original mean (SD)**				**-** **Estimated marginal mean (Standard error)** **-**		**Estimated marginal mean difference (Group H minus Group C) (95% CI)**	***P* value of estimated marginal mean**
	**Group H**		**Group C**		**Group H**	**Group C**		
	**n**	**Mean (SD)**	**n**	**Mean (SD)**				
2^nd^ hr	25	273.36±30.65	25	135.37±14.10	273.36 (5.22)	135.37 (4.87)	137.99 (124.26 to 151.72)	< 0.001^#^
6^th^ hr	25	281.04±32.84	25	135.13±17.41	281.04 (5.22)	135.13 (4.87)	145.91 (132.18 to 159.64)	< 0.001^#^
12^th^ hr	25	280.68±35.97	25	137.18±13.33	280.68 (5.22)	137.18 (4.87)	143.50 (129.77 to 157.23)	< 0.001^#^
24^th^ hr	25	276.96±31.84	25	136.38±15.23	276.96 (5.22)	136.38 (4.87)	140.58 (126.85 to 154.31)	< 0.001^#^

Data are shown as mean±SD; Group C, conventional oxygen therapy; Group H, high frequency nasal oxygenation; CI, confidence interval; PaO_2_, partial pressure of oxygen; *P *< 0.05, statistically significant^#^; hr, hour; SD, standard deviation

**Table 4. Comfort Score and Complications Between the Groups:** 

**Variable name**	**Group H (n = 25)**	**Group C (n = 25)**	***P *value**
Comfort score median [IQR]	4.0 (3.5 to 4.0)	3.0 (2.0 to 3.0)	<0.001^#^
Complications associated with intervention	5 (20.0)	4 (16.0)	0.733
Chest X-ray proven atelectasis at the end of 3^rd^ day	2 (8.0)	1 (4.0)	1.00
Proportion of PPCs	2 (8.0)	1 (4.0)	1.00

Group C, conventional oxygen therapy; Group H, high frequency nasal oxygenation; IQR, interquartile range; *P *< 0.05, statistically significant^#^; PPCs, postoperative pulmonary complications
